# Regulatory Effect of Resveratrol on Inflammation Induced by Lipopolysaccharides *via* Reprograming Intestinal Microbes and Ameliorating Serum Metabolism Profiles

**DOI:** 10.3389/fimmu.2021.777159

**Published:** 2021-11-15

**Authors:** Sujuan Ding, Hongmei Jiang, Jun Fang, Gang Liu

**Affiliations:** College of Bioscience and Biotechnology, Hunan Agricultural University, Hunan Provincial Engineering Research Center of Applied Microbial Resources Development for Livestock and Poultry, Changsha, China

**Keywords:** resveratrol, inflammation, serum metabolites, intestinal microbes, DSS

## Abstract

The purpose of this study was to explore the regulatory effect of resveratrol (RES) on lipopolysaccharide (LPS)-induced inflammation and its influence on intestinal microorganisms and serum atlas in murine models during the development of inflammation to explore a novel method for the regulation of inflammation. Mice were randomly assigned to three groups: control (CON), LPS, and RES–LPS. The results showed that RES mitigated the inflammatory damage to the intes-tines and liver induced by LPS. Compared with the LPS group, RES treatment decreased the levels of TNF-α, IL-6, IFN-γ, myeloperoxidase, and alanine aminotransferase in the liver. Serum metabolic profile monitoring showed that, compared with the CON group, LPS decreased the levels of five metabolites, including cycloartomunin and glycerol triundecanoate, and increased the levels of eight metabolites, including N-linoleoyl taurine and PE(O-16:0/20:5(5Z), 8Z, 11Z, 14Z, 17Z). Conversely, RES treatment increased the levels of eight metabolites, including pantothenic acid, homovanillic acid, and S-(formylmethyl)glutathione, and reduced seven metabolites, including lysoPE(20:4(8Z,11Z,14Z,17Z)/0:0) and 13-cis-retinoic acid, etc., in comparison with the LPS group. Moreover, RES treatment alleviated the negative effects of LPS on intestinal microbes by reducing, for instance, the relative abundance of Bacteroidetes and Alistipes, and increasing the relative abundance of Lactobacillus. These results suggest that RES has great potential for preventing in-flammation.

## Introduction

Resveratrol (RES) is a class of stilbene polyphenols with trans 3,5,4’-trihydroxy that has various properties, including anticancer, antioxidant, and anti-inflammatory properties (1–3). Inflammation is a fundamental aspect of gastrointestinal function. Since dietary antigens and toxins produced by microorganisms abound in the gastrointestinal tract, a healthy digestive tract is considered to be in a constant “controllable” inflammation state. The occurrence of inflammation is closely related to the occurrence of diseases (4–6). For instance, celiac disease is related to inflammation and oxidative stress, which are caused by an increase in reactive oxygen species and a decrease in antioxidant capacity (7). *In vitro* evidence shows that RES reduces the expression of COX-2 in intestinal cells stimulated by lipopolysaccharides (LPS). Moreover, it inhibits the transport of the NF-κB p65 subunit from the cytoplasm to the nucleus, which is related to the inhibition of IκBα phosphorylation and degradation, and downregulates COX-2 and PGE2 to achieve negative regulation of IKK phosphorylation in intestinal cells (8). RES can also inhibit the growth of the SK-ChA-1 cell line, increase the activity of lactate dehydrogenase and alkaline phosphatase, and interfere with the cell cycle, specifically accumulation in the G1/S phase, which indicates that it has an antitumor effect (9).

Despite the protection provided by the intestinal mucosal barrier, LPS may still reach the liver through the hepatic portal vein (10). The main role of the liver is to absorb nutrients from the portal vein and distribute them to other organs. The liver is also the main organ involved in the detoxification of LPS (11, 12). A previous study demonstrated that RES inhibited the expression of interleukin (IL)-1β, tumor necrosis factor (TNF) α, IL-12, and aryl hydrocarbon receptor (Ahr) induced by LPS, as well as endogenous eicosanoid production in the liver cells of Atlantic salmon (13). Another study showed that piceatannol (a derivative of RES) reduced liver oxidative stress induced by LPS, as well as the levels of TNF-α, IL-1β, and IL-6 in the liver, suggesting that it could be used as a drug for preventing acute liver failure by preventing inflammation and oxidative stress (14). However, studies on the role of RES in intestinal or hepatic inflammation are limited. Therefore, the purpose of this study was to investigate whether RES can regulate inflammation and improve serum metabolism and intestinal microorganisms in murine models of inflammation induced by LPS.

## Materials and Methods

### Animal and Experimental Protocol

The Hunan Agricultural University’s Animal Ethics Committee granted approval for the animal procedures used in this study. Eight-week-old male ICR mice (21 ± 1 g) were kept under a 12-hour light and dark cycle at 22 ± 2°C with 40–60% humidity. The mice were randomly assigned to three groups (*n* = 8): a control (CON) group, an LPS group, and a RES–LPS group. The mice in the CON group received intraperitoneal saline injections. The mice in the RES–LPS group received RES (100 mg.kg^−1^ of body weight) intragastrically for seven days before receiving an intraperitoneal injection of LPS (L2880; Sigma-Aldrich). The mice in the LPS group received intraperitoneal LPS (15 mg.kg^-1^ of body weight) at 9 a.m., and other mice were injected with normal saline intraperitoneally. After 24 h of LPS treatment, blood was collected from the orbital veins of all mice, which were subsequently sacrificed by cervical dislocation. The animals’ livers were then extracted. Also, a small opening was made in the middle part of the colon using sterilized medical scissors, and about 2 g of contents were collected with a sterilized medicine spoon.

The blood samples were centrifuged at 3,000 rpm for 10 min to obtain serum, which was stored at −80°C until analysis. The livers were rinsed in ice phosphate-buffered saline and stored at −80°C. Small pieces of liver and jejunum were cut and fixed in 4% paraformaldehyde. The colonic contents were divided into sterile cryopreserve tubes, which were quickly placed in liquid nitrogen for quick-freezing and stored at −80°C until analysis.

### Intestinal Histomorphology

Pieces of freshly isolated jejunum tissue were fixed in 4% paraformaldehyde at room temperature for more than 24 h. Usually, 5-μm specimen sections were embedded in paraffin and stained with hematoxylin and eosin at room temperature for 10 min. The pathological status of intestinal tissue was examined under a light microscope (Olympus BX41; Olympus, Münster, Germany), and images were captured. The degree of intestinal tissue damage was graded according to the degree of inflammation as previously described (15, 16). In brief, the severity of histological inflammation was scored as 0, none; 1, mild; 2, moderate; and 3, severe. Inflammatory cell infiltration was scored as 0, normal; 1, mucosal; 2, submucosal; and 3, osmotic transmural expansion. Epithelial lesions were scored as 0, complete; 1, crypt structure deformation; 2, erosion; and 3, ulcers. The extent of lesions was scored as 0, none; 1, 2, multifocal; and 3, spread. Edema was scored as 0, none; 1, mild mucosal; 2, submucosal; and 3, mucosal). The scores were added to obtain an overall intestinal tissue damage score.

### Immune Factor Measurements

An enzyme-linked immunosorbent assay kit (Jiangsu Yutong Biological Technology Co., Ltd., Jiangsu, China) was used to detect the levels of TNF-α, IL-1β, IL-6, monocyte chemoattractant protein-1 (MCP-1), macrophage inflammatory protein-2 (MIP-2), interferon-gamma (IFN-γ), and myeloperoxidase (MPO) in the liver tissue samples according to the manufacturer’s protocol. The levels of nitric oxide (NO), alanine aminotransferase (ALT), and aspartate aminotransferase (AST) were measured using a kit according to the manufacturer’s instructions (Nanjing Jiancheng Bioengineering Institute, Nanjing, China). The levels of immune factors were expressed as protein content per milligram. The protein content in the liver tissue samples was determined using a BCA protein concentration determination kit (Beyotime, Shanghai, China).

### Serum Metabolomics Analysis

The metabolomic analysis was based on our previous study (17). Serum samples (100 μl) were placed in a 1.5-ml centrifuge tube, and 400 μl of methanol–acetonitrile solvent (1:1) was added. After ultrasonic treatment in a water bath at 4°C for 10 min, the supernatant was centrifuged at 12,000 rpm for 15 min after being kept at −20°C for 1 h. The supernatant was removed, and nitrogen was blown in ice bath. The extract was redissolved in 80% methanol and filtered using a 0.22-μm microporous filter (Jinteng Co. Ltd., Tianjin, China). It was then analyzed using an Agilent 6545 Q-TOF LC/MS system (Agilent Technologies Co., Ltd., China). Agilent Profinder was used to correct the retention time, identify, extract, integrate, and align the peak, and finally output in CEF format. Statistical processing was performed using Agilent Massive Parallel. The Human Metabolome Database (HMDB; http://www.hmdb.ca/) and MassBank (http://www.massbank.jp) were used to search for and infer the structures of possible biomarkers. Variables with significant differences between the two groups were assessed using a *t*-test. CAMERA was used to remove adduct, isotope, or fragment ions and identify metabolites with significant differences.

### 16S Ribosomal RNA Amplicon Sequencing for Colon Microbes

A method of 16S rRNA sequencing in colon microbes was used as previously described (16). In brief, genomic DNA quality control, design and synthesis of primer splices, polymerase chain reaction (PCR) amplification, PCR product purification, quantification, and homogenization, and MiSeq high-throughput sequencing (Illumina, USA) were performed.

### Data Analysis

One-way analysis of variance (ANOVA) was used to assess the homogeneity of variance using Levene’s test and then Student’s *t*-test. The analyses were performed using IBM SPSS Statistics 21 (IBM Corp., Armonk, NY, USA) for Windows. Prism 7 (GraphPad Software, San Diego, CA, USA) was used to investigate the potential relationship between intestinal microbes and serum metabolites. The Pearson correlation coefficient was used to assess the correlations between colonic microbes and differential serum metabolites. The level of statistical significance was set to *p* < 0.05.

## Results

### Regulatory Effect of Resveratrol on Intestinal Damage Induced by Lipopolysaccharides

**Figure 1:**
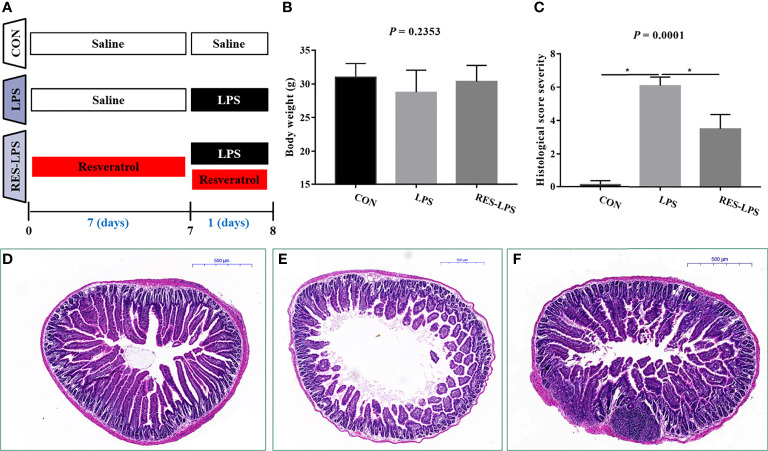
Effect of RES on intestinal inflammation induced by LPS. **(A)** Time arrangement and treatment of this experiment. **(B)** Final body weights of the mice in the three groups. **(C)** Histopathological severity scores of the three groups. Effects of RES on intestinal histology in the CON **(D)**, LPS **(E)**, and RES–LPS **(F)** groups, *p < 0.05.

The mice received RES for seven days and were then intraperitoneally injected with LPS. The animals’ final weights were recorded and analyzed (**Figure 1A**). The administration of LPS resulted in weight loss. However, RES prevented the negative effect of LPS on body weight to a certain extent (**Figure 1B**; *p* > 0.05). Moreover, histopathological analysis of intestinal tissue samples showed that LPS caused shortening of jejunal villi and infiltration of inflammatory cells, which mainly included macrophages, neutrophils, and lymphocytes (**Figure 1E**). No lesions were observed in the intestinal tissue samples of the CON group (**Figure 1D**). RES mitigated the intestinal damage caused by LPS (**Figure 1F**). The histopathological scores were consistent with the histologic examination (**Figure 1C**; *p* < 0.05).

### Regulatory Effect of Resveratrol on Liver Injury Induced by Lipopolysaccharides

**Figure 2:**
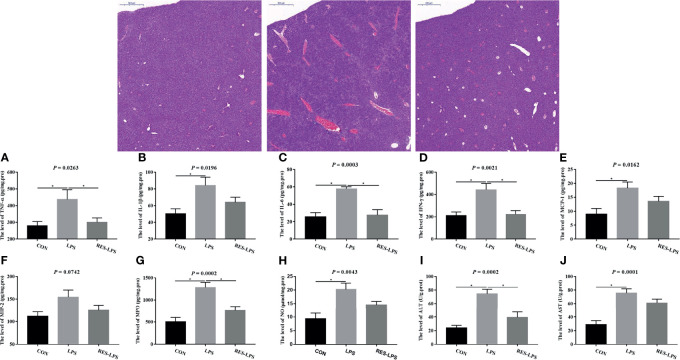
Effects of RES on liver injury and inflammation induced by LPS. **(A–J)** Levels of TNF-α, IL-1β, IL-6, IFN-γ, MCP-1, MCP-2, MPO, NO, ALT, and AST in the three groups. Liver histopathology images of tissue samples of the CON, LPS, and RES–LPS groups (hematoxylin and eosin staining; ×100). *p < 0.05.

The liver tissue injury observations revealed that LPS treatment resulted in greater central lobular necrosis, hyperplasia, and liver inflammation compared to the CON group, whereas RES mitigated LPS-induced damage to the liver (**Figure 2**). Moreover, the levels of TNF-α, IL-6, IFN-γ, MPO, and ALT in the RES–LPS group were significantly lower than in the LPS group (*p* < 0.05) and comparable to those in the CON group (**Figures 2A, C, D, G, I**). Furthermore, the levels of IL-1β, MCP-1, NO, and AST in the LPS group were significantly higher than in the CON group. The values in the RES–LPS group were lower than in the LPS group, although the differences were not statistically significant (**Figures 2B, E, F, H, J**; *p* > 0.05).

### Resveratrol Affected the Serum Metabolic Profile During Intestinal Inflammation Induced by Lipopolysaccharides

**Figure 3:**
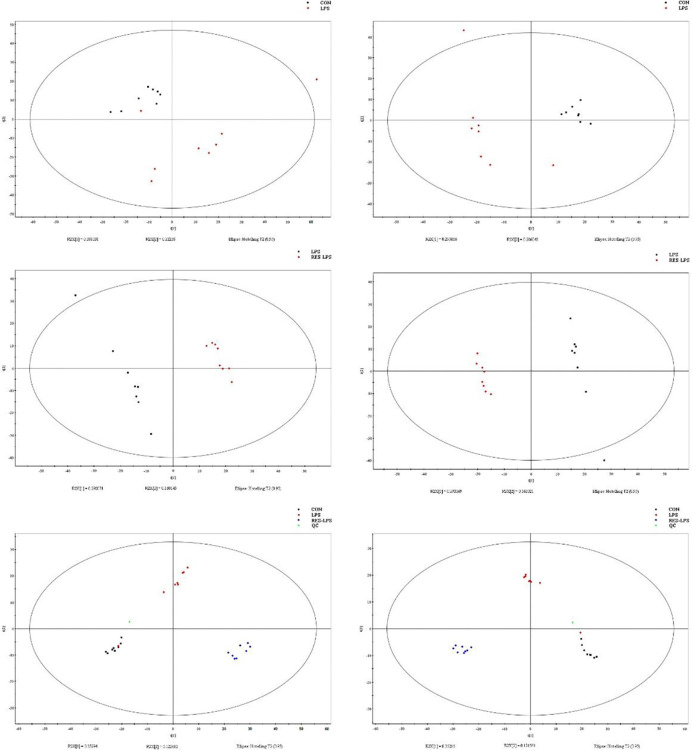
Score plots of principal component analysis and PLS-DA models with their corresponding R2X and T2 values. Score plots between two and three groups.

Principal component analysis was performed using Umetrics (Sweden) to determine similarities between spectral profiles (**Figure 3**). Each serum sample scattergram revealed the distribution of positive and negative ions in a section model. To further evaluate differences in the abundance of metabolites between the samples, PLS-DA, a supervised multidimensional statistical method, was used (**Figure 3**). The differential metabolites were identified and screened according to the variable importance in the projection (VIP) and *p*-values in the PLS-DA model—that is, VIP values greater than 1.5 and *p*-values less than 0.01. Thirteen metabolites were screened between the CON and LPS groups. Among them, cycloartomunin, (+)−tephropurpurin, glycerol triundecanoate, neoglucobrassicin, and cyclopassifloic acid E were downregulated, while N-linoleoyl taurine, N-valerylglycine methyl ester, tetrahydrocortisol, PE(O-16:0/20:5(5Z,8Z,11Z,14Z,17Z)), 7-oxo-11E-tetradecenoic acid, fructoselysine, fenothiocarb sulfoxide, and 4-keto myristic acid were upregulated. Moreover, 15 differential metabolites were screened between the LPS and RES–LPS groups. Among them, pantothenic acid, 3-hydroxy valeric acid, homovanillic acid, 3-hydroxydodecanoic acid, lysoPE(0:0/16:0), PE(22:6(4Z,7Z,10Z,13Z,16Z,19Z)/0:0), PS(P-16:0/13:0), and S-(formylmethyl)glutathione were downregulated, whereas PE(18:1(11Z)/22:6(4Z,7Z,10Z,13Z,16Z,19Z)), lysoPE(20:4(8Z,11Z,14Z,17Z)/0:0), pubescenol, 13-cis-retinoic acid, PE(22:6(4Z,7Z,10Z,13Z,16Z,19Z)/0:0), 2-dodecylbenzenesulfonic acid, and cholesterol sulfate were upregulated (**Tables 1**, **2**).

**Table 1:** Differential metabolites identified in serum samples of the CON and LPS groups.

Name	Mass	Retention time (min)	Formula	KEGG ID	VIP	Fold change	Regulation (CON *vs.* LPS)	*P* Value
Cycloartomunin	448.152	18.79	C26H24O7	–	1.695	1.33	Up	1.12×10^-5^
(+)-Tephropurpurin	424.1525	19.59	C24H24O7	–	1.687	1.36	Up	1.29×10^-5^
Glycerol triundecanoate	596.499	21.55	C36H68O6	–	1.679	1.12	Up	1.07×10^-5^
Neoglucobrassicin	524.0771	19.69	C17H22N2O10S2	C08424	1.644	4.88	Up	5.3×10^-5^
Cyclopassifloic acid E	552.365	20.14	C31H52O8	–	1.620	1.49	Up	4.7×10^-5^
N-linoleoyl taurine	387.2439	16.20	C20H37NO4S	–	1.646	-5.21	Down	2.14×10^-5^
N-Valerylglycine methyl ester	173.105	6.50	C8H15NO3	–	1.619	-1.05	Down	4.02×10^-5^
Tetrahydrocortisol	366.2404	9.17	C21H34O5	C05472	1.613	-1.04	Down	4.59×10^-5^
PE [O-16:0/20:5(5Z,8Z,11Z,14Z,17Z)]	723.5188	18.79	C41H74NO7P	–	1.563	-1.06	Down	1.46×10^-4^
7-oxo-11E-Tetradecenoic acid	240.1723	13.20	C14H24O3	–	1.560	-1.17	Down	1.27×10^-4^
Fructoselysine	308.16	13.20	C12H24N2O7	C16488	1.555	-1.46	Down	1.39×10^-4^
Fenothiocarb sulfoxide	329.1298	9.91	C13H19NO3S	C11085	1.537	-1.52	Down	1.91×10^-4^
4-keto myristic acid	242.1879	14.25	C14H26O3	–	1.537	-1.05	Down	1.98×10^-4^

**Table 2:** Differential metabolites identified in serum samples of the LPS and RES–LPS groups.

Name	Mass	Retention time (min)	Formula	KEGG ID	VIP	Fold change	Regulation (LPS *vs.* RES-LPS)	P Value
Pantothenic Acid	219.1103	2.63	C9H17NO5	C00864	1.732	-19.67	Down	1.72×10^-8^
3-hydroxy valeric acid	118.063	3.47	C5H10O3	–	1.707	-17.50	Down	5.86×10^-8^
Homovanillic acid	182.0577	4.18	C9H10O4	C05582	1.676	-17.74	Down	1.92×10^-6^
3-Hydroxydodecanoic acid	216.1723	13.00	C12H24O3	–	1.638	-16.44	Down	6.95×10^-6^
LysoPE(0:0/16:0)	453.2857	14.50	C21H44NO7P	–	1.629	-20.26	Down	9.34×10^-6^
PE[22:6(4Z,7Z,10Z,13Z,16Z,19Z)/0:0]	525.2859	13.61	C27H44NO7P	–	1.628	-20.53	Down	9.73×10^-6^
PS(P-16:0/13:0)	723.4642	19.92	C35H68NO9P	–	1.590	-15.08	Down	2.64×10^-5^
S-(Formylmethyl)glutathione	349.0946	5.94	C12H19N3O7S	C14871	1.575	-14.56	Down	3.51×10^-5^
PE[18:1(11Z)/22:6(4Z,7Z,10Z,13Z,16Z,19Z)]	789.5331	19.59	C45H76NO8P	C00350	1.703	15.39	Up	7.02×10^-8^
LysoPE[20:4(8Z,11Z,14Z,17Z)/0:0]	501.2859	13.61	C25H44NO7P	–	1.687	20.00	Up	1.47×10^-6^
Pubescenol	584.2621	21.01	C32H40O10	–	1.681	17.72	Up	1.59×10^-6^
13-cis-retinoic acid, Isotretinoin	300.2087	14.63	C20H28O2	D00348	1.636	15.22	Up	7.66×10^-6^
PE[22:6(4Z,7Z,10Z,13Z,16Z,19Z)/0:0]	525.2865	13.91	C27H44NO7P	–	1.629	22.05	Up	9.30×10^-6^
2-Dodecylbenzenesulfonic acid	326.1911	18.78	C18H30O3S	–	1.597	13.94	Up	2.11×10^-5^
Cholesterol sulfate	466.3118	18.78	C27H46O4S	C18043	1.574	13.79	Up	3.63×10^-5^

### Resveratrol Ameliorates Intestinal Microbes Induced by Lipopolysaccharides

**Figure 4:**
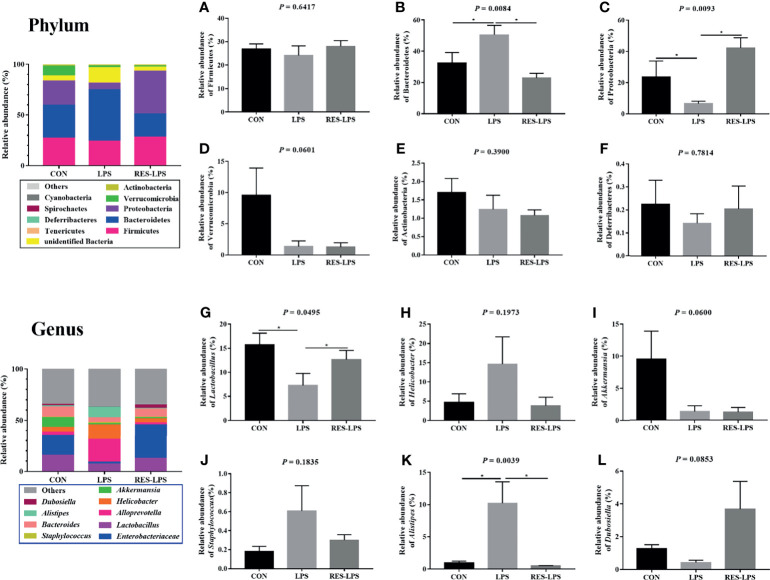
Effects of RES on intestinal microbes during the development of inflammation induced by LPS. Relative abun-dances of the phyla Firmicutes **(A)**, Bacteroidetes **(B)**, Actinobacteria **(C)**, Verrucomicrobia **(D)**, Actinobacteria **(E)**, and Deferribacteres **(F)**. Relative abundances of the genera *Lactobacillus* **(G)**, *Helicobacter* **(H)**, *Akkermansia* **(I)**, *Staphylococcus* **(J)**, *Alistipes* **(K)**, and *Dubosiella* **(L)** in the three groups. *p < 0.05.

**Figure 5:**
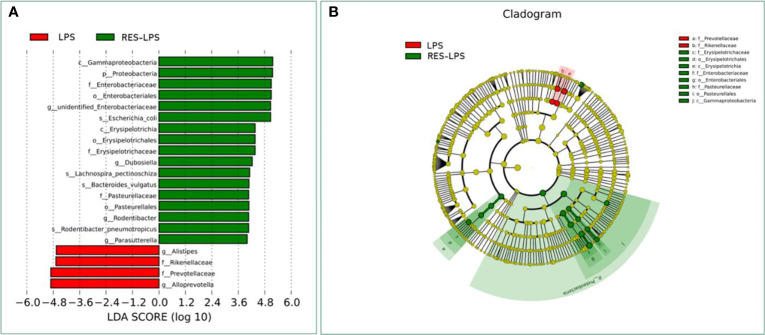
Computational LDA **(A)** score and LEfSe **(B)** analysis comparing LPS and RES-LPS group.

**Figure 6:**
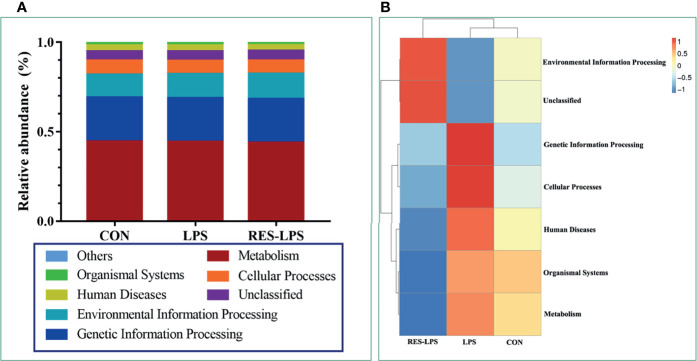
Effects of RES on intestinal microbial function. According to the annotation results of KEGG database, Tax4Fun functional annotation relative abundance histogram **(A)** for the top 10 functions and functional annotation clustering heat map **(B)** were made among CON, LPS and RES-LPS groups.

The composition of and changes in gut microbes are closely related to the development of diseases. To explore the role of RES in the regulation of gut microbes during the development of inflammation induced by LPS, 16S rRNA high-throughput sequencing was used to detect the composition and abundance of intestinal microorganisms in feces. At the phylum level, the dominant bacteria were Firmicutes, Bacteroidetes, and Proteobacteria. The overall proportions of these three phyla in the CON, LPS, and RES–LPS groups were 92.95%, 82.63%, and 94.32%, respectively. The proportions of Firmicutes were 26.93%, 24.15%, and 28.09%, respectively. The proportions of Bacteroidetes were 32.65%, 50.5%, and 22.86%, respectively. The proportions of Proteobacteria were 23.78%, 6.61%, and 42.12%, respectively. The relative abundance of Bacteroidetes was significantly lower in the RES–LPS than in the LPS group (**Figures 4B, C**; *p* < 0.05). At the genus level, LPS treatment reduced the relative abundance of *Lactobacillus* and increased the relative abundance of *Alistipes*, while RES prevented this negative effect (**Figures 4G, K**; *p* < 0.05). Furthermore, linear discriminant analysis (LDA) and effect size (LEfSe) analysis showed that *Lactobacillus* differed significantly between the LPS and RES–LPS groups (LDA > 3.0; *p* < 0.05; **Figures 5A, B**). There was no significant difference in other microorganisms (**Figures 4A, D–F, H–J, L**). Subsequently, Tax4fun was used to annotate the 16S rRNA gene sequences extracted from the KEGG database, and functional annotation information was obtained. The results showed that these functions were mainly concentrated in pathways related to metabolism, genetic information processing, and cellular processes and that there were differences between the LPS group and the CON and RES–LPS groups (**Figures 6A**), while LPS treatment affects microbial functions (**Figures 6B**).

### Correlation Analysis of Differential Microbes and Serum Metabolites

**Figure 7:**
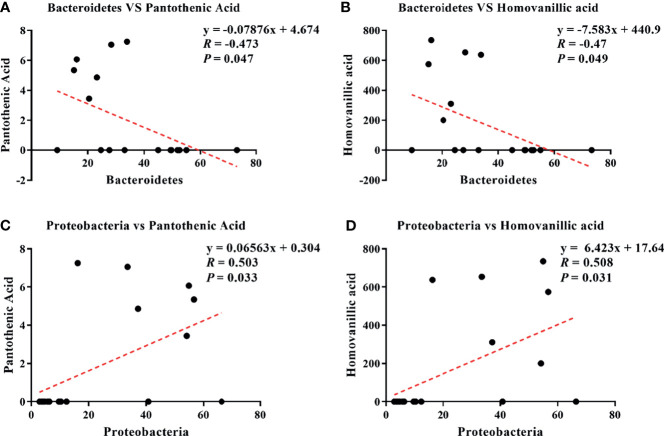
Correlations between differential microbes and serum metabolites during the development of inflammation induced by LPS: Bacteroidetes–pantothenic acid **(A)**, Bacteroidetes–homovanillic acid **(B)**, Proteobacteria–pantothenic acid **(C)**, and Proteobacteria–homovanillic acid **(D)**.

Pearson correlation analysis is a statistical analysis method used to measure the correlation between two variables (16, 18). The results showed four correlations: Bacteroidetes–pantothenic acid (*r* = −0.473; *p* = 0.047; **Figure 7A**), Bacteroidetes–homovanillic acid (*R* = −0.47; *p* = 0.049; **Figure 7B**), Proteobacteria–pantothenic acid (*R* = 0.503; *p* = 0.033; **Figure 7C**), and Proteobacteria–homovanillic acid (*R* = 0.508; *p* = 0.031; **Figure 7D**).

## Discussion

Inflammation is a usually “uncontrollable” immune response to stimuli that trigger the activation of innate and adaptive immunity, such as pathogens or tissue damage. Therefore, inflammation is a pivotal driver of many pathological conditions, such as cancer, inflammatory bowel disease, liver failure, and Alzheimer’s disease (19, 20). Despite continuous advances in research, due to the complexity and particularity of the disease, many studies are still needed to explore new potential drugs. Polyphenols are a group of compounds worthy of exploration due to their great therapeutic potential.

RES is an organic non-flavonoid polyphenol compound known for its important pharmacological properties, including antioxidant, anti-diabetic, and anti-inflammatory properties (21). This study demonstrates that RES can mitigate intestinal and hepatic injury induced by LPS, reduce the levels of TNF-α, IL-6, IFN-γ, MPO, and ALT in liver tissue, promote the production of anti-inflammatory and antioxidant metabolites in serum, increase the relative abundance of *Lactobacillus* in the intestinal tract, and improve intestinal microbial function during the development of inflammation induced by LPS.

Intestinal inflammation is an important indicator of gastrointestinal function. When the body is in a healthy state, the intestinal tract is in a continuous and controllable inflammatory state (22). Polyphenols are phytochemicals commonly found in plants that exert immunomodulatory activity and provide intestinal microecological stability (23, 24). RES is a natural polyphenol derived from berries and other fruits and has few side effects (25). A previous study showed that RES significantly improved the functions of mitochondria and antiviral CD8 while promoting the synthesis of cytokines and improving the activation of MT antioxidants in T cells (26). Another study found that RES suppressed the expression of pro-inflammatory cytokines, decreased the severity of intestinal inflammation during the development of IBD, and inhibited the production of reactive oxygen species and neutrophil infiltration (27). Furthermore, RES has been shown to directly target innate and acquired immune cells, such as macrophages, dendritic cells, and lymphocytes (28, 29). Animal model experiments have also shown that RES could play an immunomodulatory role by reducing the expression of CD28 and CD80 receptors and increasing the expression of IL-10 (30).

The liver is an important detoxification organ and participates in the detoxification of LPS. It is therefore vital to maintain its health (10, 31). A previous study showed that 100 or 200 M of RES could inhibit the expression of IL-1, TNF, COX2, and Ahr in liver cells in an LPS-induced inflammatory state (13). In our study, RES mitigated LPS-induced hepatic and intestinal injury and inflammatory cell infiltration and reduced the expression of liver immune factors, indicating that it could alleviate LPS-induced inflammation.

The intestinal microbiota is a complex micro-ecosystem composed of many microorganisms that closely interact with the host. It directly or indirectly participates in the functional regulation of the body, affecting the host’s health, including defense, nutritional metabolism, and immune regulation (20, 32, 33). Certain bacteria are closely related to inflammatory factors that affect tissue inflammation. For example, increased abundance of *Precotella*, which is associated with T helper (Th) type 17-mediated mucosal inflammation enhancement, can increase the production of Th-17 polarized cytokines, such as IL-1 and IL-23, by activating Toll-like receptor 2 (34). *Lactobacillus* is considered the main candidate for probiotics aimed at preventing uncontrolled intestinal inflammation and has great potential to protect against IBD (35). A previous study reported that *Lactobacillus reuteri* inactivated NF-κB in HT-29 cell lines by blocking IκB degradation and preventing p65 nuclear transfer (36). Our study showed that LPS stimulation reduced the relative abundance of *Lactobacillus* in the intestine, while RES prevented its reduction.

Metabolomics can reveal the characteristic chemical fingerprints left by cells in the process of functioning, providing evidence that can explain biological processes (37, 38). The results of this study showed that in LPS-induced inflammation, RES increased the levels of serum metabolites such as cholesterol sulfate, 2-dodecylbenzenesulfonic acid, 13-cis-retinoic acid, and lysoPE(20:4(8Z,11Z,14Z,17Z)/0:0). Cholesterol sulfate is a vital sterol sulfate in human plasma. It can participate in the development of a barrier by inducing the transcription of gene-encoding transglutaminase I, which is an essential protein crosslinking enzyme for barrier formation (39).

Overall, this study provides the basis for investigating the potential of RES to relieve inflammation. Specifically, the histopathological analysis revealed that RES alleviated LPS-induced intestinal and hepatic tissue damage and inflammatory cell infiltration. Moreover, it prevented the increase in TNF-α, IL-6, IFN-γ, MPO, and ALT levels induced by LPS. Furthermore, it regulated the intestinal microbial composition and serum metabolism profiles in an inflammatory state. These results indicate that RES has the potential to be used therapeutically to reduce tissue damage and uncontrolled inflammation.

## Data Availability Statement

The datasets presented in this study can be found in online repositories. The names of the repository/repositories and accession number(s) can be found below: https://www.ncbi.nlm.nih.gov/, PRJNA763035.

## Ethics Statement

The animal study was reviewed and approved by The Hunan Agricultural University’s Animal Ethics Committee granted approval for the animal procedures used in this study.

## Author Contributions

Writing – Original draft preparation, SD and GL. Index detection – SD. Writing – Review & editing, HJ, JF, and GL. All the authors contributed to manuscript revision, read and approved the submitted version.

## Funding

This research was supported by National Natural Science Foundation of China (No. 31772642, 31672457), Local Science and Technology Development Project Guided by The Central Government (YDZX20184300002303, 2018CT5002), and Hunan Provincial Science and Technology Department (2019TP2004, 2018WK4025, 2020NK2004, 2020ZL2004), China Postdoctoral Science Foundation (2018M632963, 2019T120705), Scientific Research Fund of Hunan Provincial Education Department (2020JGYB112), and Double first-class construction project of Hunan Agricultural University (SYL201802003, YB2018007, CX20190497, CX20190524).

## Conflict of Interest

The authors declare that the research was conducted in the absence of any commercial or financial relationships that could be construed as a potential conflict of interest.

## Publisher’s Note

All claims expressed in this article are solely those of the authors and do not necessarily represent those of their affiliated organizations, or those of the publisher, the editors and the reviewers. Any product that may be evaluated in this article, or claim that may be made by its manufacturer, is not guaranteed or endorsed by the publisher.
